# Safety and efficacy of a novel four-hook needle versus hook wire for CT-guided preoperative localization of pulmonary nodules: a propensity score-matched analysis

**DOI:** 10.3389/fsurg.2026.1947750

**Published:** 2026-10-07

**Authors:** Rui Zhang, Yue Hu, Fangfang Tian, Xiaohui Zhao, Pengtao Bao, Haipeng Yu

**Affiliations:** 1 Department of Interventional Therapy, Tianjin Medical University Cancer Institute & Hospital, Tianjin, China; 2 National Clinical Research Center for Cancer, Tianjin Medical University Cancer Institute & Hospital, Tianjin, China; 3 Department of Respiratory and Critical Care Medicine, The Eighth Medical Center of Chinese PLA General Hospital, Beijing, China; 4 Department of Neurosurgery, Aviation General Hospital, Beijing, China; 5 Department of Neurosurgery, Tianjin Medical University General Hospital, Tianjin, China

**Keywords:** four-hook needle, localization, pneumothorax, pulmonary hemorrhage, pulmonary nodules

## Abstract

**Background:**

To compare the safety and effectiveness of a novel four-hook needle with conventional Hook wire for CT-guided preoperative localization of pulmonary nodules and to identify factors associated with localization-related complications.

**Methods:**

A retrospective study was conducted in patients who underwent CT-guided localization followed by video-assisted thoracoscopic surgery between January 2024 and June 2025. Propensity score matching yielded 248 matched pairs. Post-matching comparisons were performed using paired statistical methods. Generalized estimating equation models with the same reduced set of clinically relevant covariates were used for pulmonary hemorrhage and pneumothorax, with matched pair specified as the clustering variable.

**Results:**

Technical localization success was achieved in all patients. Tip-to-nodule distance was comparable between the Hook wire and four-hook needle groups (4.82 ± 4.91 mm vs. 4.74 ± 4.22 mm, *P* = 0.842). The four-hook needle shortened localization time (7.69 ± 3.18 min vs. 8.76 ± 3.22 min, *P* < 0.001). Pulmonary hemorrhage (8.1% vs. 13.7%, *P* = 0.054) and pneumothorax (10.1% vs. 16.1%, *P* = 0.067) occurred less frequently in the four-hook group, although the differences did not reach statistical significance. Marker dislodgement was significantly less frequent with the four-hook needle (0.4% vs. 3.2%, *P* = 0.039), and post-localization VAS pain scores were lower (2.56 ± 1.28 vs. 4.00 ± 1.21, *P* < 0.001). In matched-pair clustered multivariable analyses, greater distance from the pleura, greater puncture depth, and more needle adjustments were associated with pulmonary hemorrhage, while more needle adjustments and prone positioning were associated with pneumothorax.

**Conclusions:**

Both localization techniques provided accurate preoperative localization of pulmonary nodules. The four-hook needle shortened localization time, reduced marker dislodgement, and was associated with lower post-localization VAS pain scores. Pulmonary hemorrhage and pneumothorax were numerically less frequent with the four-hook needle, but these differences were not statistically significant after accounting for the matched-pair structure.

## 1 Introduction

Lung cancer remains the most frequently diagnosed cancer worldwide and the leading cause of cancer death (1). Low-dose computed tomography (LDCT) screening enables earlier detection and has been shown to reduce lung cancer mortality in high-risk populations (2–4). With the expanding use of CT-based screening and health examinations, increasing numbers of small pulmonary nodules, including ground-glass and part-solid lesions, are being detected.

Histopathological diagnosis may be obtained by percutaneous transthoracic needle biopsy; however, diagnostic performance is lower for very small lesions. Tsukada et al. reported an accuracy of 66.7% for CT-guided automated needle biopsy of nodules smaller than 1 cm (5). For selected small peripheral lesions with a high suspicion of malignancy, minimally invasive surgical resection can provide both diagnosis and definitive treatment, and contemporary studies support sublobar resection in appropriately selected early-stage tumors (6–8).

Video-assisted thoracoscopic surgery (VATS) is widely used for the minimally invasive management of pulmonary nodules, but small, deep, and subsolid lesions may be difficult to identify by visual inspection or palpation alone. Preoperative localization is therefore commonly used to facilitate accurate resection while preserving lung parenchyma. CT-guided localization techniques include Hook wire, microcoil, dyes such as indocyanine green, medical adhesive, and other emerging devices; systematic reviews and contemporary reviews indicate high overall technical success but different profiles of invasiveness, stability, and complications among methods (9–14).

Hook wire remains widely used because it is simple and technically familiar, but marker dislodgement, pneumothorax, pulmonary hemorrhage, and procedure-related discomfort are recognized limitations (15–19). Four-hook localization systems were developed to provide multidirectional anchoring and a flexible suture tail rather than a rigid external wire. Recent comparative and prospective studies have reported high localization success together with lower displacement or dislodgement rates, shorter localization time, and lower pain scores with four-hook devices in selected cohorts (20–23). Accordingly, the present study used propensity score matching (PSM) to compare a novel four-hook needle with conventional Hook wire for CT-guided preoperative localization and to explore factors associated with localization-related complications.

## 2 Materials and methods

### 2.1 Study population

This retrospective study included patients who underwent CT-guided preoperative localization of pulmonary nodules in the Department of Interventional Radiology at Tianjin Cancer Hospital between January 2024 and June 2025, followed by VATS in the Department of Thoracic Surgery. The study protocol was approved by the Institutional Ethics Committee of Tianjin Cancer Hospital. Written informed consent was obtained from all patients before the procedure after detailed explanations of the purpose, procedure, potential benefits, and possible risks. Patients also consented to the use of their clinical data for research purposes. The patient selection process is shown in Figure 1.

**Figure 1:**
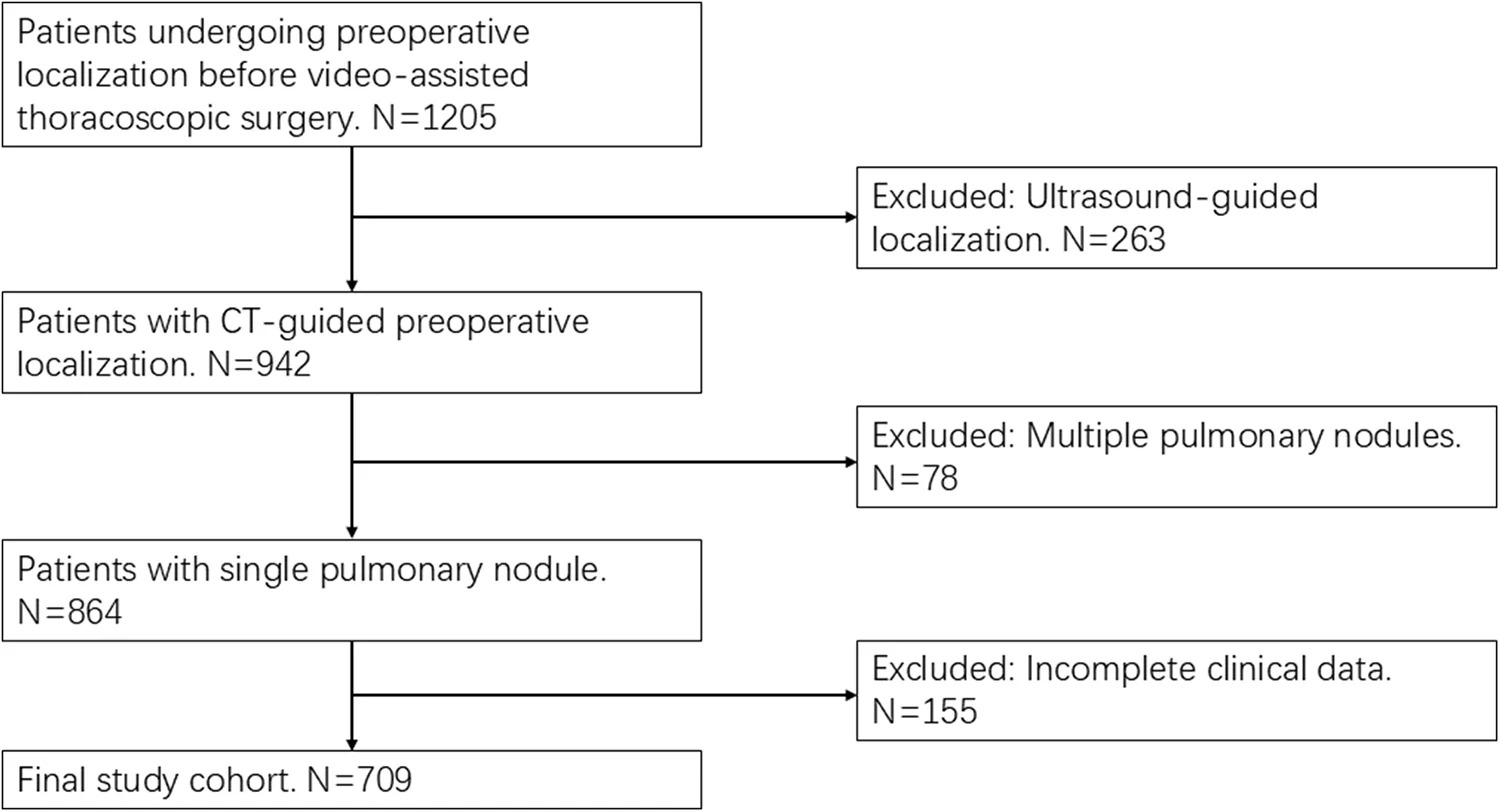
Flowchart describing the patient inclusion and exclusion procedures of the study.

### 2.2 Inclusion and exclusion criteria

#### 2.2.1 Inclusion criteria

1. Age ≥18 years;
2. Presence of a solitary pulmonary nodule with radiological features suggestive of malignancy and scheduled for surgical resection;
3. Lesions considered difficult to localize intraoperatively by visual inspection or manual palpation alone;
4. Underwent attempted CT-guided preoperative localization using either a conventional Hook wire or a four-hook needle;
5. Availability of complete clinical and imaging data.

#### 2.2.2 Exclusion criteria

1. Age <18 years;
2. Lesions that could be accurately identified intraoperatively without preoperative localization;
3. Contraindications to percutaneous puncture, including severe coagulation disorders or severe cardiopulmonary insufficiency;
4. Inability to cooperate during the localization procedure;
5. Missing or incomplete clinical data.

### 2.3 Preoperative localization procedure

#### 2.3.1 Imaging evaluation and puncture planning

All localization procedures were performed by two experienced interventional radiologists, each of whom had performed more than 100 CT-guided pulmonary puncture procedures before the study period. Both radiologists performed both Hook wire and four-hook needle localization procedures. The two localization techniques were used contemporaneously during the study period. The same CT scanner was used for both techniques throughout the study, with experienced radiologic technologists assisting the procedures. CT acquisition, puncture planning, local anesthesia, localization, and immediate post-localization CT assessment followed the same general institutional workflow for both groups. Preprocedural CT images were independently reviewed by experienced interventional radiologists. The puncture pathway was determined according to lesion size, anatomical location, distance from the pleura, and surrounding structures. The shortest and safest trajectory was preferentially selected while avoiding interlobar fissures, pulmonary bullae, and major vascular structures whenever possible. Patient positioning (supine, prone, or lateral decubitus) was determined based on lesion location and the planned puncture route.

#### 2.3.2 Localization procedure

After routine skin disinfection and sterile draping, local anesthesia was achieved with 2% lidocaine. Under CT guidance, the localization needle was advanced along the predetermined trajectory to a position adjacent to the target nodule. Patients in the Hook wire group underwent localization using a conventional single-hook wire device (20G*100 mm, PAJUNK, Germany), whereas patients in the four-hook group underwent localization using a novel four-hook needle system (SS510-10, Senscure, China) (Figure 2).

**Figure 2:**
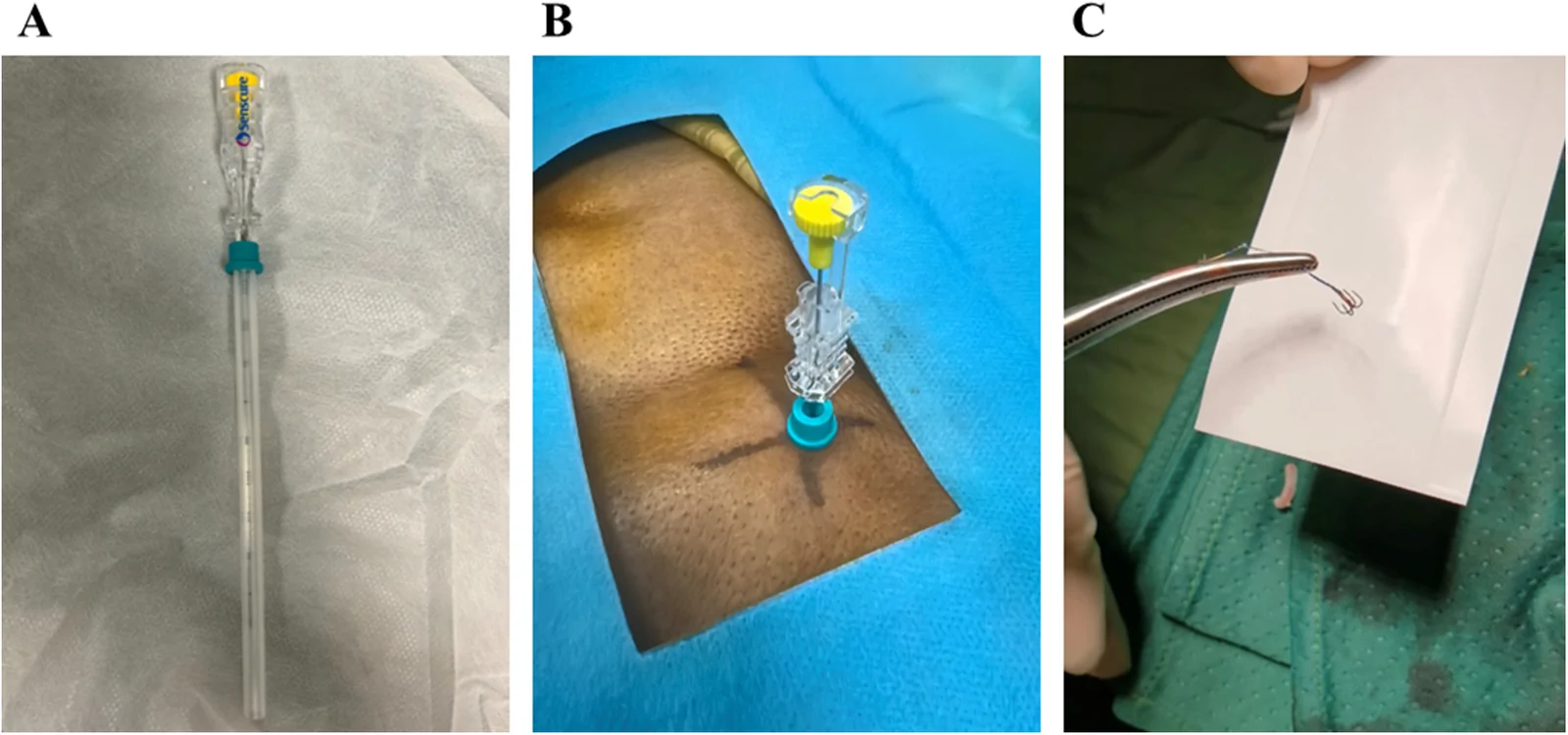
**(A)** general structure of four-hook needle. **(B)** Imaging showing lung localization via four-hook needle. **(C)** Four-hook needle retrieved after lesion resection.

Following deployment of the localization device, repeat CT scans were obtained to confirm needle position and to evaluate immediate procedure-related complications, including pneumothorax and pulmonary parenchymal hemorrhage. Tip-to-nodule distance and puncture depth were recorded (Figure 3).

**Figure 3:**
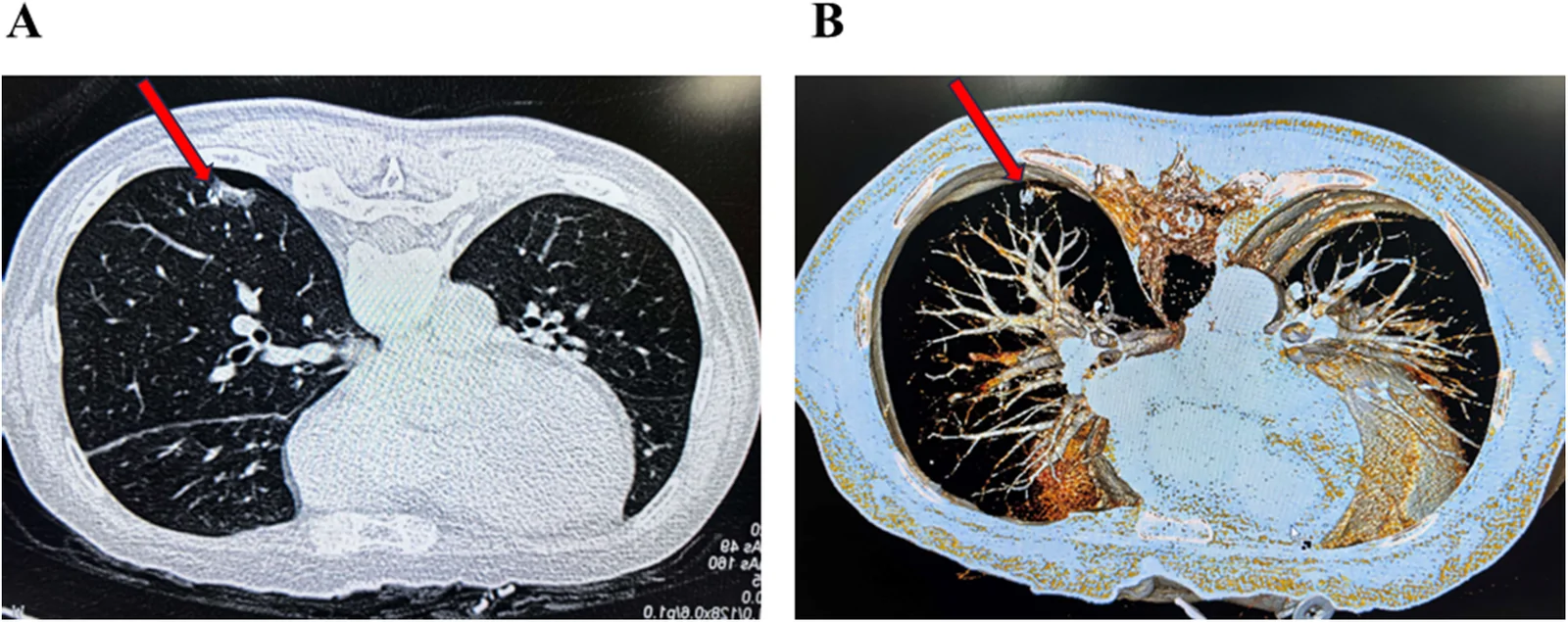
**(A)** post-procedural CT scans of four-hook needle localization. **(B)** 3D reconstructed CT images. The red arrow points to the pulmonary nodule after four-hook needle localization.

#### 2.3.3 Procedural assessment

The number of needle adjustments and total localization time (defined as the interval between the initial puncture and confirmation of successful localization on the final CT scan) were documented. Marker dislodgement was recorded as a localization-related technical event.

#### 2.3.4 Thoracoscopic resection

After localization, patients were transferred directly to the operating room for VATS. The extent of surgical resection, including wedge resection, segmentectomy, or lobectomy, was determined according to intraoperative frozen-section pathological findings (Figure 4).

**Figure 4:**
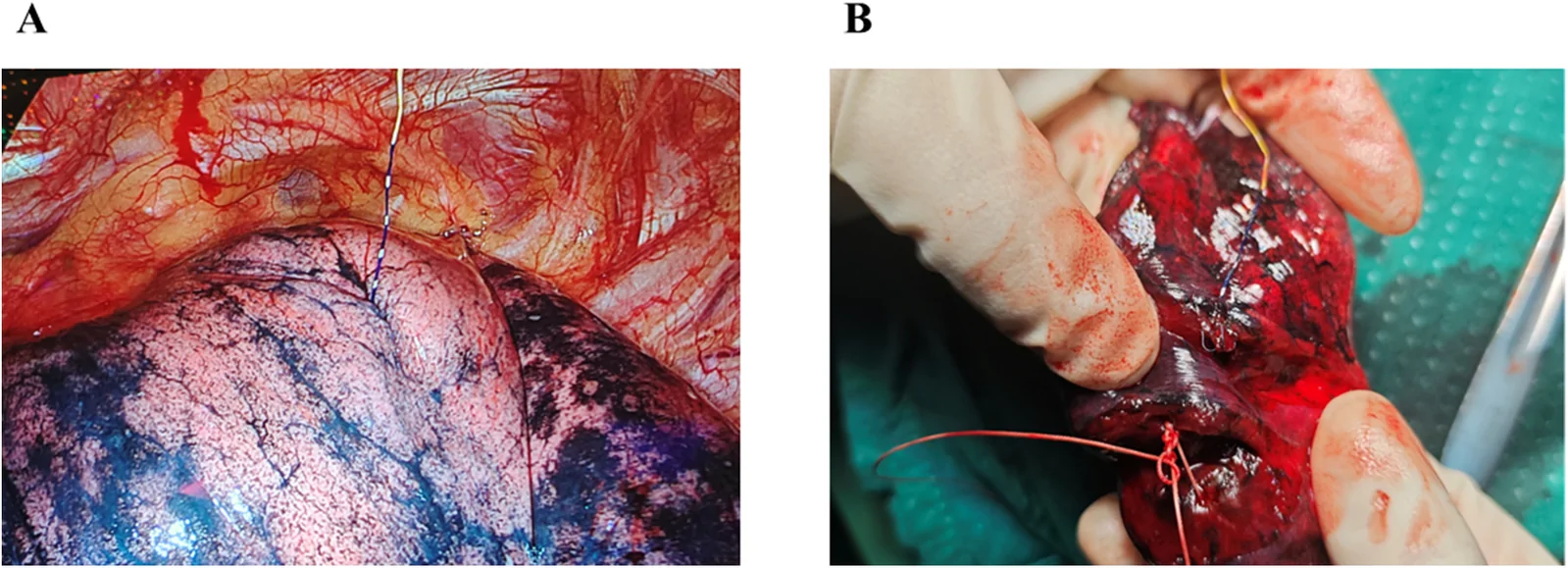
Thoracoscopic visualization of the four-hook needle during pulmonary nodule resection. **(A)** Thoracoscopic view showing the four-hook needle penetrating the lung parenchyma. **(B)** The resected pulmonary specimen with the four-hook needle and localized pulmonary nodule.

### 2.4 Data collection

Demographic and clinical variables included sex, age, smoking history, duration of disease, tumors or other pulmonary comorbidities, anticoagulant use, and family history. Imaging and lesion characteristics included nodule location, radiological characteristics, distance from the pleura, and maximum nodule diameter. Procedure-related variables included localization method, puncture depth, patient position, tip-to-nodule distance, number of needle adjustments, localization time, marker dislodgement, pulmonary hemorrhage, pneumothorax, pleural reaction, post-localization VAS pain score, and hospital stay.

### 2.5 Outcome definitions

Technical localization success was defined as successful deployment of the localization device adjacent to the target nodule, confirmation of its position on post-localization CT, and subsequent identification and resection of the target lesion during thoracoscopic surgery without repeat localization because of localization failure. Localization accuracy was quantitatively assessed as the shortest distance between the deployed needle tip and the target nodule on the final post-localization CT images. Pain intensity was assessed using a 0–10 visual analogue scale (VAS) after completion of CT-guided localization and before VATS. The score therefore represented post-localization rather than postoperative pain and was obtained before general anesthesia, thoracic surgical resection, chest-tube placement, and postoperative analgesic management. Both localization techniques were performed under local anesthesia with 2% lidocaine. The VAS score was self-reported by the patient and recorded by the medical staff.

Pulmonary hemorrhage and pneumothorax were assessed on immediate post-localization CT. Pulmonary hemorrhage was defined as newly developed ground-glass opacity or consolidation along the needle tract or adjacent to the target lesion, whereas pneumothorax was defined as newly developed air within the pleural space. All radiologically detected pulmonary hemorrhage and pneumothorax events were recorded, irrespective of clinical symptoms or the need for therapeutic intervention.

Marker dislodgement was defined as displacement or detachment of the deployed localization marker from its intended anchoring position before or during thoracoscopic resection. Pleural reaction was defined as a transient vagal reflex syndrome induced by procedural stimulation of the parietal pleura, occurring during or immediately after localization. Localization time was defined as the interval from the initial puncture to confirmation of successful localization on the final CT scan.

### 2.6 Propensity score matching

As treatment allocation was not randomized in this retrospective study, differences in baseline characteristics could have influenced the choice of localization device and subsequent comparisons between the two groups. PSM was used to make the groups more comparable with respect to measured baseline characteristics. Variables entered into the propensity score model were selected before outcome analysis on the basis of clinical relevance. The variables included sex, age, duration of disease, smoking history, tumors or other pulmonary comorbidities, anticoagulant use, family history, nodule location, radiological characteristics, distance from the pleura, and maximum nodule diameter. Propensity scores for four-hook needle localization were estimated using logistic regression. Patients were matched 1:1 using nearest-neighbor matching without replacement, with a caliper of 0.05 applied to the raw propensity score. Covariate balance before and after matching was assessed using standardized mean differences (SMDs), with an absolute SMD of 0.10 used as a reference for adequate balance. Propensity-score distributions were examined to assess overlap between the two groups.

### 2.7 Statistical analysis

Statistical analyses were performed using SPSS version 27.0 (IBM Corp., Armonk, NY, USA) and Python with the statsmodels package. Propensity score matching and descriptive/post-matching comparative analyses were conducted in SPSS, whereas multivariable generalized estimating equation (GEE) models and related model-checking and sensitivity analyses were performed using statsmodels in Python. Continuous variables are presented as mean ± standard deviation (SD), and categorical variables as frequencies and percentages.

Because the post-matching cohort consisted of 248 individually matched pairs, post-matching comparisons accounted for the matched-pair structure. Continuous outcomes were compared using paired-samples t tests or Wilcoxon signed-rank tests, as appropriate. Binary categorical outcomes were compared using McNemar's test, and multicategory categorical outcomes were compared using the Bhapkar test.

Multivariable analyses of pulmonary hemorrhage and pneumothorax were performed using generalized estimating equations (GEE) with a binomial distribution and logit link. Matched pair was specified as the clustering variable, and an exchangeable working correlation structure with robust standard errors was used to account for within-pair correlation. There were 54 pulmonary hemorrhage events and 65 pneumothorax events in the matched cohort. To limit model complexity relative to the number of events and to provide a consistent adjustment strategy, the same reduced set of clinically relevant variables was included in both models: localization method, tumors or other pulmonary comorbidities, distance from the pleura, puncture depth, number of needle adjustments, and patient position. No significance-based variable-selection procedure was used, and localization method was retained as the primary exposure of interest irrespective of statistical significance. Patient position was treated as a categorical variable, with the supine position as the reference category.

There were no missing values for the outcomes or covariates included in the multivariable models; therefore, no imputation was required. Linearity of continuous predictors on the logit scale was assessed as part of model checking, and sensitivity analyses were performed when evidence of nonlinearity was identified. Multicollinearity was assessed using variance inflation factors. Odds ratios for distance from the pleura and puncture depth are reported per 1-mm increase, whereas the odds ratio for the number of needle adjustments is reported per one additional adjustment. Adjusted odds ratios (ORs), 95% confidence intervals (CIs), and *P* values are reported for all variables included in the models. A two-sided *P* value <0.05 was considered statistically significant.

## 3 Results

### 3.1 Baseline characteristics

Before matching, 709 patients were included, with 259 in the Hook wire group and 450 in the four-hook needle group. No patients were excluded from propensity score estimation because of missing data in the variables used for matching. After 1:1 matching, 248 matched pairs (496 patients) were retained. Eleven patients in the Hook wire group and 202 in the four-hook needle group remained unmatched.

Baseline balance improved after matching. The largest absolute standardized mean difference (SMD) decreased from 0.258 before matching to 0.105 after matching. Most covariates had an absolute SMD below 0.10 in the matched cohort; a small residual difference remained for family history (SMD = 0.105). The propensity-score distributions showed greater overlap after matching. Baseline characteristics and balance measures before and after matching are presented in Table 1, with additional matching diagnostics shown in Figure 5.

**Table 1:** Comparison of baseline characteristics between the hook-wire group and four-hook needle group before and after PSM.

Variables	Before PSM					After PSM				
	Total	Hook wire	Four-hook needle	P	SMD before	Total	Hook wire	Four-hook needle	P	SMD after
Sex, *n* (%)				0.03*	0.232				0.642	0.042
Female	484 (68.3%)	159 (61.4%)	325 (72.2%)			311 (62.7%)	153 (61.7%)	158 (63.7%)		
Male	225 (31.7%)	100 (38.6%)	125 (27.8%)			185 (37.3%)	95 (38.3%)	90 (36.3%)		
Age (Mean ± SD)	54.48 ± 11.84	54.14 ± 12.29	54.68 ± 11.59	0.552	0.046	54.42 ± 11.69	54.58 ± 12.13	54.26 ± 11.25	0.762	0.027
Duration of disease, Month (Mean ± SD)	12.93 ± 17.80	12.52 ± 17.48	13.17 ± 18.00	0.636	0.037	12.89 ± 18.08	12.80 ± 17.76	12.98 ± 18.44	0.914	0.010
Smoking history, *n* (%)				<0.01*	0.258				0.755	0.028
Yes	149 (21.0%)	72 (27.8%)	77 (17.1%)			123 (24.8%)	63 (25.64%)	60 (24.2%)		
No	560 (79.0%)	187 (72.2%)	373 (82.9%)			373 (75.2%)	185 (74.6%)	188 (75.8%)		
Tumors or other pulmonary comorbidities, *n* (%)				0.216	0.098				0.811	0.022
Yes	129 (18.2%)	41 (15.8%)	88 (19.6%)			84 (16.9%)	41 (16.5%)	43 (17.3%)		
No	580 (81.8%)	218 (84.2%)	362 (80.4%)			412 (83.1%)	207 (83.5%)	205 (82.7%)		
Anticoagulants, *n* (%)				0.418	0.063				0.296	0.094
Yes	97 (13.7%)	39 (15.1%)	58 (12.9%)			68 (13.7%)	38 (15.3%)	30 (12.1%)		
No	612 (86.3%)	220 (84.9%)	392 (87.1%)			428 (86.3%)	210 (84.7%)	218 (87.9%)		
Family history, *n* (%)				0.172	0.105				0.243	0.105
Yes	63 (8.9%)	28 (10.8%)	35 (7.8%)			39 (7.9%)	23 (9.3%)	16 (6.5%)		
No	646 (91.1%)	231 (89.2%)	415 (92.2%)			457 (92.1%)	225 (90.7%)	232 (93.5%)		
Nodule location, *n* (%)				0.480	0.130				0.784	0.086
Left upper lobe	185 (26.1%)	65 (25.1%)	120 (26.7%)		0.036	134 (27.0%)	64 (25.8%)	70 (28.2%)		0.055
Left lower lobe	139 (19.6%)	49 (18.9%)	90 (20.0%)		0.027	95 (19.2%)	47 (19.0%)	48 (19.4%)		0.010
Right upper lobe	226 (31.9%)	85 (32.8%)	141 (31.3%)		0.032	166 (33.5%)	81 (32.7%)	85 (34.3%)		0.034
Right middle lobe	36 (5.1%)	18 (6.9%)	18 (4.0%)		0.130	29 (5.8%)	17 (6.9%)	12 (4.8%)		0.086
Right lower lobe	123 (17.3%)	42 (16.2%)	81 (18.0%)		0.047	72 (14.5%)	39 (15.7%)	33 (13.3%)		0.069
Radiological characteristics, *n* (%)				0.012*	0.227				0.784	0.055
GGN	577 (81.4%)	196 (75.7%)	381 (84.7%)		0.227	389 (78.4%)	193 (77.8%)	196 (79.0%)		0.029
SSN	100 (14.1%)	48 (18.5%)	52 (11.6%)		0.196	81 (16.3%)	43 (17.3%)	38 (15.3%)		0.055
SN	32 (4.5%)	15 (5.8%)	17 (3.8%)		0.094	26 (5.2%)	12 (4.8%)	14 (5.6%)		0.036
Distance from the pleura, mm (Mean ± SD)	10.26 ± 9.26	10.92 ± 9.41	9.87 ± 9.16	0.146	0.113	10.74 ± 9.47	11.30 ± 9.59	10.18 ± 9.33	0.189	0.053
Maximum nodule diameter, mm (Mean ± SD)	9.36 ± 3.18	9.07 ± 3.15	9.52 ± 3.18	0.071	0.141	9.22 ± 3.18	9.22 ± 3.12	9.23 ± 3.24	0.989	0.001

PSM: Propensity Score Matching, SMD, standardized mean difference; SD, Standard Deviation, GGN, Ground-glass nodule, SSN, Subsolid nodule, SN, Solid nodule.

* *P* < 0.05.

**Figure 5:**
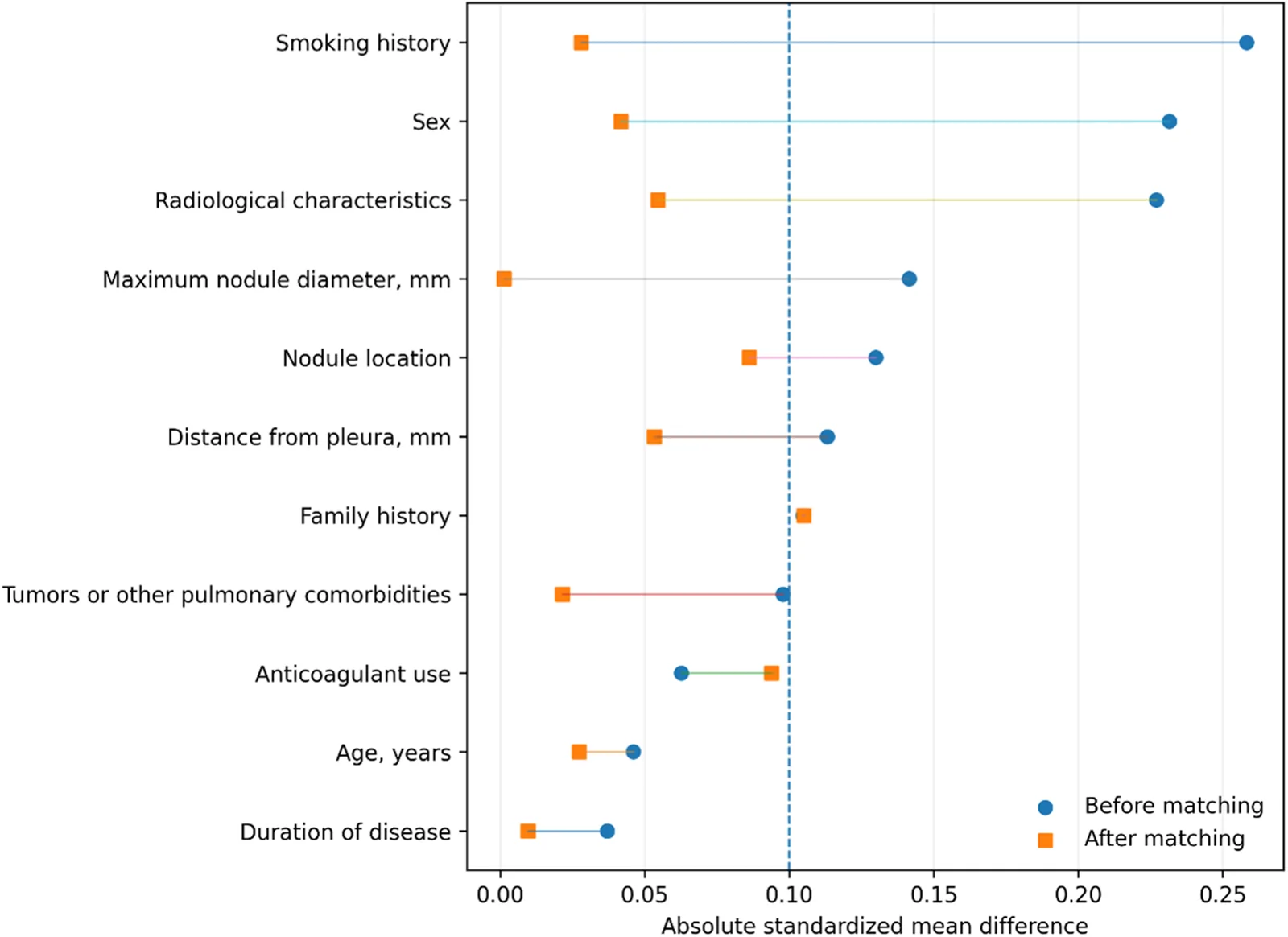
Absolute standardized mean differences (SMDs) are shown for the covariates included in the propensity score model before and after matching. The dashed line indicates an absolute SMD of 0.10.

### 3.2 Localization performance

A total of 709 CT-guided localization procedures were attempted during the study period. Technical localization was successfully completed in all cases according to the predefined criteria, and no patient was excluded because of localization failure. No pulmonary hemorrhage or pneumothorax event required chest-tube drainage, emergency surgery, or other invasive intervention, and no localization-related complication resulted in postponement of the planned VATS procedure. In the matched cohort, localization accuracy was comparable between the two techniques. The mean tip-to-nodule distance was 4.82 ± 4.91 mm in the Hook wire group and 4.74 ± 4.22 mm in the four-hook needle group (*P* = 0.842). The number of needle adjustments was also comparable (1.57 ± 1.14 vs. 1.44 ± 0.87, *P* = 0.147). Localization time was significantly shorter in the four-hook needle group than in the Hook wire group (7.69 ± 3.18 min vs. 8.76 ± 3.22 min, *P* < 0.001) (Table 2).

**Table 2:** Comparison of surgery-related data between hook wire group and four-hook group.

Variables	Hook wire group	Four-hook needle group	P
Tip-to-nodule distance, mm	4.82 ± 4.91	4.74 ± 4.22	0.842
Adjustment times	1.57 ± 1.14	1.44 ± 0.87	0.147
Procedure time, min	8.76 ± 3.22	7.69 ± 3.18	<0.001*
Pathological type, *n* (%)			0.666
Invasive adenocarcinoma	181 (73.0%)	188 (75.8%)	
Adenocarcinoma *in situ*	19 (7.7%)	20 (8.1%)	
Chronic inflammation	25 (10.1%)	17 (6.9%)	
Other types	23 (9.3%)	23 (9.3%)	
Patient position, *n* (%)			0.867
Supine position	131 (52.8%)	134 (54.0%)	
Lateral position	19 (7.7%)	16 (6.5%)	
Decubitus position	98 (39.5%)	98 (39.5%)	
Surgical procedure, *n* (%)			0.807
Wedge resection	186 (75.0%)	181 (73.0%)	
Segmentectomy	58 (23.4%)	64 (25.8%)	
Lobectomy	4 (1.6%)	3 (1.2%)	
Pulmonary parenchymal hemorrhage, *n* (%)			0.054
No	214 (86.3%)	228 (91.9%)	
Yes	34 (13.7%)	20 (8.1%)	
Pneumothorax, *n* (%)			0.067
No	208 (83.9%)	223 (89.9%)	
Yes	40 (16.1%)	25 (10.1%)	
Pleural reaction, *n* (%)			0.648
No	236 (95.2%)	239 (96.4%)	
Yes	12 (4.8%)	9 (3.6%)	
Hook dislodgement, *n* (%)			0.039*
No	240 (96.8%)	247 (99.6%)	
Yes	8 (3.2%)	1 (0.4%)	
Post-localization VAS pain score	4.00 ± 1.21	2.56 ± 1.28	<0.001*
Hospital stay	7.05 ± 1.84	7.03 ± 1.98	0.903

VAS: Visual Analogue Scale.

* *P* < 0.05.

### 3.3 Pathological findings and surgical treatment

Postoperative pathological evaluation demonstrated a similar distribution of lesion types in the two matched groups. Invasive adenocarcinoma accounted for the majority of cases, followed by adenocarcinoma *in situ*, chronic inflammatory lesions, and other pathological entities. No significant difference in pathological composition was observed between groups (*P* = 0.666) (Table 2).

Most patients underwent wedge resection, with segmentectomy and lobectomy performed less frequently. The distribution of surgical procedures was comparable between the matched groups (*P* = 0.807) (Table 2).

### 3.4 Localization-related complications

Pulmonary hemorrhage occurred in 13.7% of patients in the Hook wire group and 8.1% in the four-hook needle group. Although the incidence was numerically lower with the four-hook needle, the difference did not reach statistical significance in the matched-pair analysis (*P* = 0.054). Pneumothorax showed a similar pattern, occurring in 16.1% and 10.1% of patients, respectively (*P* = 0.067). Marker dislodgement was significantly less frequent in the four-hook needle group than in the Hook wire group (0.4% vs. 3.2%, *P* = 0.039). Pleural reaction was uncommon in both groups, with no significant difference (3.6% vs. 4.8%, *P* = 0.648) (Table 2).

### 3.5 Post-localization pain and hospital stay

Post-localization VAS pain scores were significantly lower in the four-hook needle group than in the Hook wire group (2.56 ± 1.28 vs. 4.00 ± 1.21, *P* < 0.001). Hospital stay was comparable between the two groups (7.03 ± 1.98 vs. 7.05 ± 1.84 days, *P* = 0.903) (Table 2).

### 3.6 Risk factors for pulmonary hemorrhage

In the matched-pair clustered GEE model, greater distance from the pleura was associated with pulmonary hemorrhage (OR = 1.050 per 1-mm increase, 95% CI: 1.025–1.076, *P* < 0.001). Greater puncture depth was also associated with pulmonary hemorrhage in the primary linear model (OR = 1.019 per 1-mm increase, 95% CI: 1.004–1.033, *P* = 0.011), as was a higher number of needle adjustments (OR = 1.423 per additional adjustment, 95% CI: 1.095–1.848, *P* = 0.008). A possible nonlinear association between puncture depth and pulmonary hemorrhage was identified during model checking and was examined in sensitivity analysis. Localization method was not independently associated with pulmonary hemorrhage (four-hook vs. Hook wire: OR = 0.604, 95% CI: 0.331–1.101, *P* = 0.100). Tumors or other pulmonary comorbidities showed a borderline association (OR = 1.893, 95% CI: 0.976–3.670, *P* = 0.059). Neither lateral decubitus nor prone positioning was significantly associated with pulmonary hemorrhage compared with the supine position (Table 3). An example of pulmonary parenchymal hemorrhage after localization is shown in Figure 6.

**Figure 6:**
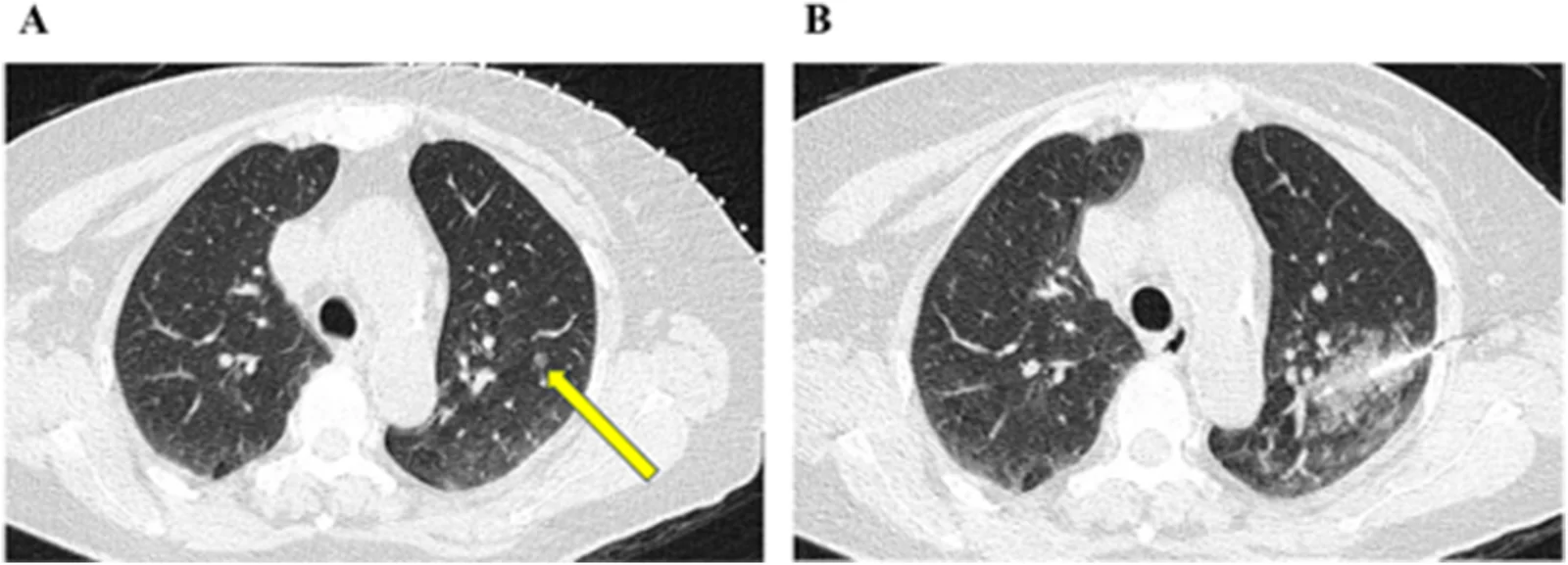
**(A)** Pre-localization CT images of ground-glass nodule in the left upper lobe. **(B)** Post-procedural follow-up CT revealed hemorrhage adjacent to the nodule.

**Table 3:** Reduced matched-pair clustered multivariable analysis of pulmonary hemorrhage.

Variables	OR	95%CI	P
Localization method: four-hook vs. Hook wire	0.604	0.331–1.101	0.100
Tumors/other pulmonary comorbidities	1.893	0.976–3.670	0.059
Distance from pleura, per 1-mm increase	1.050	1.025–1.076	<0.001*
Puncture depth, per 1-mm increase	1.019	1.004–1.033	0.011*
Needle adjustments, per additional adjustment	1.423	1.095–1.848	0.008*
Patient position (Ref: supine)			
Lateral decubitus	1.101	0.368–3.293	0.864
Prone	1.096	0.562–2.136	0.788

ORs for distance from the pleura and puncture depth are expressed per 1-mm increase. The OR for needle adjustments is expressed per one additional adjustment. Supine positioning was used as the reference category.

* *P* < 0.05.

### 3.7 Risk factors for pneumothorax

In the matched-pair clustered GEE model, a higher number of needle adjustments was associated with pneumothorax (OR = 1.599 per additional adjustment, 95% CI: 1.237–2.067, *P* < 0.001). Prone positioning was also associated with pneumothorax compared with the supine position (OR = 1.752, 95% CI: 1.013–3.030, *P* = 0.045), whereas the association for lateral decubitus positioning was of borderline statistical significance (OR = 2.436, 95% CI: 0.978–6.065, *P* = 0.056). Localization method was not independently associated with pneumothorax (four-hook vs. Hook wire: OR = 0.641, 95% CI: 0.368–1.115, *P* = 0.116). Tumors or other pulmonary comorbidities, distance from the pleura, and puncture depth were not significantly associated with pneumothorax (Table 4).

**Table 4:** Reduced matched-pair clustered multivariable analysis of pneumothorax.

Variables	OR	95%CI	P
Localization method: four-hook vs. Hook wire	0.641	0.368–1.115	0.116
Tumors/other pulmonary comorbidities	0.882	0.434–1.795	0.730
Distance from pleura, per 1-mm increase	1.016	0.989–1.045	0.247
Puncture depth, per 1-mm increase	0.995	0.980–1.010	0.535
Needle adjustments, per additional adjustment	1.599	1.237–2.067	<0.001*

Patient position (Ref: supine)			
Lateral decubitus	2.436	0.978–6.065	0.056
Prone	1.752	1.013–3.030	0.045*

ORs for distance from the pleura and puncture depth are expressed per 1-mm increase. The OR for needle adjustments is expressed per one additional adjustment. Supine positioning was used as the reference category.

* *P* < 0.05.

## 4 Discussion

The increasing use of LDCT has shifted lung cancer detection toward smaller and earlier lesions and has increased the clinical relevance of pulmonary nodule management (2–4, 10). For small or ground-glass–dominant lesions selected for surgery, accurate limited resection is increasingly important (6–8). Because deep or nonpalpable nodules may be difficult to identify during VATS, CT-guided localization has become an important adjunct, with multiple device- and material-based approaches currently available (9, 24).

Propensity score matching improved comparability between the two localization groups, yielding 248 matched pairs from the 709 included patients. Both Hook wire and four-hook needle localization achieved a 100% technical success rate, and tip-to-nodule distance was comparable between groups. These findings indicate that the four-hook device provides localization accuracy comparable to conventional Hook wire. Systematic reviews of preoperative CT-guided pulmonary nodule localization have likewise reported high technical success across commonly used localization methods (14, 25). The principal advantages of the four-hook needle in the present cohort therefore appear to relate more to procedural efficiency, fixation stability, and patient comfort than to improved targeting accuracy.

One of the main findings was the shorter localization time with the four-hook needle. The number of needle adjustments was numerically lower in the four-hook group, although the difference was not statistically significant. This may be related to the multidirectional anchoring configuration, which can provide stable fixation after deployment without leaving a rigid metal wire protruding from the chest wall. Several studies of four-hook or other anchored localization devices have similarly reported shorter localization time, lower displacement or dislodgement rates, or improved fixation-related outcomes compared with conventional wire-based localization (20–23, 26, 27).

Pulmonary hemorrhage and pneumothorax were numerically less frequent in the four-hook needle group than in the Hook wire group, but the paired comparisons did not reach conventional statistical significance. In contrast, marker dislodgement remained significantly less frequent with the four-hook needle (0.4% vs. 3.2%, *P* = 0.039). This finding is consistent with studies showing lower displacement or dislodgement rates with anchored or four-hook devices than with conventional Hook wire (15, 20–22, 26). Published CT-guided localization series report substantial variation in pneumothorax and pulmonary hemorrhage rates according to device, lesion characteristics, puncture technique, and outcome definitions (16, 17, 21, 28, 29). Thus, the clearest device-related advantage demonstrated by our matched analysis was improved fixation stability, whereas any reduction in pulmonary hemorrhage or pneumothorax requires confirmation in larger prospective studies.

Post-localization VAS pain scores were significantly lower in the four-hook needle group than in the Hook wire group. Importantly, VAS was assessed after completion of the localization procedure and before VATS; therefore, the observed difference was not attributable to the subsequent thoracic surgical procedure, general anesthesia, chest-tube placement, or postoperative analgesic management. Both groups underwent localization using the same local anesthetic protocol with 2% lidocaine. Previous comparative studies have also reported lower pain scores with four-hook or anchored devices than with conventional Hook wire (20, 22, 26), while a dedicated Hook-wire study identified puncture-related factors as determinants of moderate-to-severe post-localization pain (30). In the present study, the four-hook group required numerically fewer needle adjustments, although the difference was not statistically significant after matching. In addition, the four-hook design may provide greater flexibility in patient positioning and puncture trajectory. These procedural characteristics could potentially reduce repeated tissue and pleural stimulation and thereby contribute to lower discomfort. However, pain perception is multifactorial, and the present retrospective study was not designed to establish the mechanism underlying the observed difference in VAS scores. Hospital stay was similar between groups, suggesting that overall recovery duration was influenced more strongly by subsequent surgical management than by the localization device itself.

To further explore factors associated with localization-related complications while limiting model complexity, the same reduced set of clinically relevant covariates was entered into the GEE models for pulmonary hemorrhage and pneumothorax. Greater distance from the pleura and greater puncture depth were associated with pulmonary hemorrhage. Model checking suggested that the association with puncture depth may not be strictly linear across the entire observed range; therefore, its per-millimeter OR should be interpreted as an overall average association. Greater pleural distance and deeper intrapulmonary needle trajectories may increase the opportunity for parenchymal or vascular injury, while repeated needle manipulation may further increase tissue trauma. Consistent with this interpretation, a contemporary microcoil-versus-Hook-wire study identified greater needle penetration depth as a risk factor for pulmonary hemorrhage (16), and Kawanaka et al. reported a higher hemorrhage rate in deeply located than in shallow nodules after CT-guided lipiodol marking (31). Patient position was not independently associated with pulmonary hemorrhage in our model.

For pneumothorax, a higher number of needle adjustments was associated with increased risk, supporting careful trajectory planning and accurate initial needle placement. Prone positioning was also associated with pneumothorax compared with the supine position, whereas the association for lateral decubitus positioning was borderline. Patient position has been linked to outcomes of CT-guided pulmonary puncture, but published findings are not uniform. In a study specifically examining localization needles and posture, Wang et al. reported fewer total localization-related complications in the prone group than in the supine and lateral groups, although prone positioning was associated with more pain and decoupling (26). Prospective multicenter biopsy data have likewise shown that pneumothorax risk is influenced by procedural and patient-related factors (32). Because position in our study was selected according to lesion location and puncture route rather than randomized, the observed association should be interpreted cautiously and not as evidence of a direct causal effect.

Navigation-assisted localization may further reduce dependence on repeated manual needle adjustment. Laser-guided and robotic-assisted CT localization studies have reported improvements in procedural precision or efficiency (18, 33). More recently, a prospective study of a robotic-assisted optical navigation system reported 100% localization success, a median of zero needle adjustments, a mean targeting deviation of 1.49 mm, and localization-related complications in 8.39% of targets (34). These developments suggest that future pulmonary nodule localization may increasingly combine stable anchoring devices with image-guided navigation and trajectory planning.

## 5 Limitations

Several limitations should be acknowledged. First, this was a retrospective single-center study, and residual confounding cannot be completely excluded despite propensity score matching and matched-pair clustered analyses. Although both localization techniques were used contemporaneously, both experienced interventional radiologists performed both procedures, and the same CT equipment and general procedural workflow were maintained throughout the study period, residual confounding related to calendar time or operator-specific practice remains possible. Second, the numbers of pulmonary hemorrhage and pneumothorax events were limited. We therefore used the same reduced set of clinically relevant covariates in both multivariable models to limit model complexity and provide a consistent adjustment strategy; nevertheless, the regression findings should be interpreted as exploratory and require external validation. Third, patient position was selected according to lesion location and puncture planning rather than randomly assigned, which limits causal interpretation of the observed association between prone positioning and pneumothorax. Fourth, all procedures were performed at a high-volume cancer center, which may limit generalizability. Finally, long-term clinical outcomes and cost-effectiveness were not evaluated. Prospective multicenter studies are needed to confirm these findings.

## 6 Conclusions

In conclusion, both Hook wire and four-hook needle localization provided reliable guidance for thoracoscopic resection of pulmonary nodules. Compared with conventional Hook wire, the four-hook needle shortened localization time, reduced marker dislodgement, and was associated with lower post-localization VAS pain scores. Pulmonary hemorrhage and pneumothorax occurred numerically less frequently with the four-hook needle, although these differences did not reach statistical significance after accounting for the matched-pair structure. These findings support the four-hook needle as an effective alternative for preoperative pulmonary nodule localization. Whether the observed differences in pulmonary hemorrhage and pneumothorax are directly attributable to the localization device requires further prospective validation.

## Data Availability

The raw data supporting the conclusions of this article will be made available by the authors, without undue reservation.
